# Statistical Report of Expenditure of 109 London Hospitals

**Published:** 1919-11-22

**Authors:** 


### KING EDWARD'S HOSPITAL FUND FOR LONDON.

### Statistical Report of Expenditure of 109 London Hospitals.

The familiar annual Blue Book of King Edward's Hospital Fund for London deals this year with 109 institutions, including one more than in last year's return. Practically the whole of the hospitals of the Metropolis are dealt with, including some particulars of St. Bartholomew's and the Cancer, which, although not coming within the fold of the King's Fund, supply statistics for the annual return. With those of these two hospitals the figures represent the bearings of expenditure upon 11,566 beds in average daily occupation in 1918, and attendances in the out-patient department of 1,203,573 patients. Of the 145,212 in-patients, passing through the hospitals 27,044 were naval and military patients treated by understanding with the authorities. In reference to the last-named class we have already dealt fairly fully with their incidence upon hospital statistics.

The total expenditure of the hospitals, including St. Bartholomew's and the Cancer, in reference to every heading except capital expenditure, interest on borrowed money, and contributions to the maintenance of convalescent homes and country branches, amounted to £1,946,155. The Statistical Report does not deal with income, but it is noted, for the purposes of certain calculations, that £334,747 were paid to the hospitals by naval and military authorities.

The arrangement of the Statistical Report is much upon the same lines as in former years. In the abnormal general circumstances some features, such as particulars of laundry expenses, prices of the chief supplies, and comparisons of cost per bed between the current year and the previous year, have again been omitted.

### Is the Cheap Hospital Efficient?

The ordinary reader of a report such as this might very well ask himself why it is that one hospital should be so much more expensive to run than another, especially when each is doing the same kind of work. If the ordinary reader were well versed in hospital matters, and were aware of the extraordinary differences of standards of efficiency, he would probably approach the matter from a different direction, and, not necessarily ask why hospital A spends so much more than hospital B, but rather why hospital B spends so much less than hospital A. It is a lamentable thing that even amongst what are known as the great hospitals of the Metropolis the standard of efficiency varies so much. For one instance, all will admit that true economy in hospital work lies in making sure that the greatest good should come from the efforts of the physician, the surgeon, the nurse, or other worker, not only in their bearing upon the patient while he is within the walls of the institution but also after he has gone once more into the outer world, that he may not lose the effects of successful treatment. Yet in one of the great hospitals of London it is a fact that there is no vestige of an almoner's or after-care department, while another hospital of about the same size, and certainly in the progressive sense as great, spends several thousand pounds a year upon this useful and necessary branch of hospital work. Yet from the tables before us there is no single disclosed fact which would enable us in comparing statistics to review this important condition as to the cost of one hospital with another.

### Lively Effect of Criticism.

Criticism, favourable or unfavourable, fair or unfair, is almost always useful because it gives the criticised person an opportunity of reviewing himself or his affairs in the light of the criticism. Therefore the group comparisons, although they place side by side the efficient and the inefficient; although they yield no information as to the age or structural conditions of the building; although there is no indication (except by means of the amount spent on salaries and wages) of the number of mouths to feed in a given hospital; although the accountant of each institution has almost uncontrolled discretion in the division of the expenditure between in-patients and out-patients and as to the general arrangement of the figures; although all these conditions tend seriously to vitiate the statistics that are placed before us, they still have their uses in enabling the authorities of the compared hospitals not only to ask themselves if they are spending too much, but to discover what is often much more important, if they are spending too little.

### The Soaring Cost per Bed.

To review the whole of the figures in a critical manner, would take us beyond the limits of this article, but some interesting features may be noted in passing, especially as regards the huge rise in the cost of maintenance. We notice amongst the larger general hospitals with medical schools that the London Hospital is still highest with an annual expenditure of £174 16s. on each occupied bed, being £23 above last year's total. St. George's Hospital again comes second with £152 19s. 5d., as against £137 12s. 6d. That marvel in the realm of hospital statistics, University College Hospital, is lowest, with an average annual cost of £85 14s. 4d., being £89 1s. 8d. less than that of the London Hospital. Curiously enough University College is not lowest in the matter of the cost per out-patient.

Amongst the larger general hospitals without medical schools the cost per occupied bed ranges from £88 2s. 2d. at the Dreadnought to £122 12s. 11d. at the West London. These two hospitals had only £17 between them in 1917 and in 1918 they were separated by £34.

The previous article appeared on November 8, p. 118.

